# The Ins and Outs of Sperm Entry

**DOI:** 10.1371/journal.pbio.0040160

**Published:** 2006-04-25

**Authors:** Françoise Chanut

Egg cells have simple shapes, yet they have built-in asymmetries that can profoundly affect early steps in development. The football-shaped egg cell—or oocyte—of fruit flies contains a precisely laid mosaic of maternal molecules with the potential to induce different cell fates. As the fertilized oocyte divides, the resulting cells inherit distinct sets of maternal components, which endow them with different abilities to form head or tail, gut, muscles, or nerves. The spherical egg of frogs is also divided into areas that shift cells toward gut, muscle, or neural fate.

By contrast, the mammalian oocyte seems devoid of a clear blueprint for cell fates. All embryonic cells remain equally suited to give rise to all tissue types until implantation, when the embryo has divided several times. The general consensus is that in mammals, embryonic cells decide their fates by interacting with their neighbors, and not by a preset program handed down by the oocyte.

Still, a mammalian oocyte is not as simple as its roughly spherical shape suggests. For instance, its chromosomes hang close to its membrane, rather than at its center, and define a special area where some maternal molecules congregate. In addition, the oocyte remains in close contact with its sister cell from an earlier division, a far smaller cell called the first polar body. Both the chromosome area and the polar body are focal points that could, in theory, generate informative asymmetries. Such asymmetries may later influence which cells become the embryo versus the placental layers, or which cells initiate gastrulation movements. Watching the fertilization of mouse oocytes, Davor Solter, Takashi Hiiragi, and colleagues reported in a previous study that sperm enters the oocyte membrane preferentially in the hemisphere closest to the first polar body. A simple explanation is that the oocyte membrane is asymmetric, the hemisphere near the polar body harboring more receptors for sperm than the other. But now, after further experiments, Nami Motosugi, Solter, Hiiragi, and colleagues have come to a different conclusion.

When they removed the mouse oocytes from their protective envelope, a soft shell called the zona pellucida (ZP), the researchers found that sperm cells entered with equal frequency through both halves of the oocyte. This dispelled the notion of an asymmetrically distributed sperm receptor. The only exception was a small area lying above the oocyte's chromosomes, a taut patch of membrane that appears refractory to sperm entry.

Inside an intact ZP, the polar body presses against the oocyte, which locally increases the space between oocyte and ZP—the so-called perivitelline space (PVS). The authors reasoned that this local increase of PVS volume might be responsible for the sperm entry bias. Indeed, by simply enlarging the PVS all around the oocyte, either by removing half of the cell's content or by inserting a second polar body opposite the original one, they were able to significantly increase the frequency of sperm entry events far from the first polar body.

Using time-lapse videos and careful marking strategies, the researchers showed that sperm broke through the ZP at random locations, and could swim frantically for a few minutes before entering the oocyte. Once random motion brings a sperm cell near the polar body, the likelihood is high that it will remain there and pierce the oocyte membrane nearby, simply because of the additional swimming space afforded by the expanded PVS. This simple spatial asymmetry is sufficient, the authors argue, to explain the bias they observed in the choice of sperm entry site, without invoking any asymmetry of the oocyte membrane.

Aside from providing further evidence that the mammalian egg cell is unpatterned, these observations also support the validity of in vitro fertilization practices that introduce sperm more or less randomly directly inside the oocyte.

As to why sperm should normally spend several minutes pacing the PVS before fertilization, the authors suggest that this gives it time to complete the maturation that allows its fusion with the oocyte. That sperm should waver in the egg's antichamber before tying the knot will come as no surprise to anybody who has ever faced such a momentous decision.

## 

**Figure:**
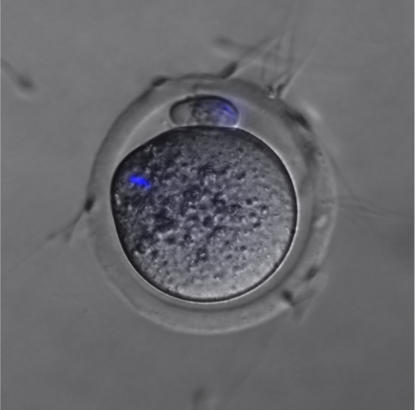
The first polar body (the smaller cell atop the oocyte) deforms the mammalian egg away from its encapsulating zona pellucida, creating a gap.

